# The erythrocyte membrane stability is associated with sleep time and social jetlag in shift workers

**DOI:** 10.1371/journal.pone.0222698

**Published:** 2019-09-23

**Authors:** Kely Raspante Cerqueira Teixeira, Luciana Alves de Medeiros, Jordane Amaral Mendes, Emília Rezende Vaz, Thúlio Marquez Cunha, Erick P. de Oliveira, Nilson Penha-Silva, Cibele Aparecida Crispim

**Affiliations:** 1 Faculty of Medicine, Federal University of Uberlândia, Uberlândia, MG, Brazil; 2 Institute of Biotechnology, Federal University of Uberlândia, Uberlândia, MG, Brazil; University of Lübeck, GERMANY

## Abstract

The osmotic stability of the erythrocyte membrane (OSEM) has been associated with changes in lipid profile, blood glucose and blood pressure. Changes in these parameters are very frequent in shift workers, possibly because of the lack of synchronization of biological rhythms, which results in the social jetlag. However, the existence of association between OSEM and circadian misalignment has not been investigated in this population. Therefore, this study investigated whether shift work, sleep time and social jetlag (SJL) are associated with biochemical and hematological variables. A population consisting of 79 men working at night (n = 37) or during the day (n = 42), aged between 21 and 65 years and with a mean BMI of 27.56 ± 4.0 kg/m^2^, was investigated cross-sectionally in relation to sleep time, SJL, anthropometric (height, weight and waist circumference) and blood variables, with emphasis on the OSEM. SJL was calculated by the absolute difference between the midpoint of sleep on work and rest days. The Generalized Linear Model (GzLM) was used to investigate the existence of associations between SJL and average sleep time in relation to the analyzed variables. Workers without SJL presented lower baseline lysis values of erythrocytes in isotonic medium in relation to workers with SJL. In addition, workers who slept on average less than 6 hours had higher OSEM, and higher total and LDL-cholesterol in relation to those who slept more than 6 hours, regardless of the shift. It is possible that the association of sleep deprivation and SJL with erythrocyte membrane stability is mediated through changes in the lipid profile.

## Introduction

Circadian rhythms are evident in virtually all biological processes, from the cellular level to the whole organism [1]. These rhythms are generated by endogenous biological clocks, with the suprachiasmatic nucleus being the major pacemaker in mammals [2]. There are also biological clocks generating circadian rhythms in peripheral tissues, as is the case of the liver, adipose tissue, heart and skeletal muscle. Also, stimuli such as body temperature, light/dark cycles, fasting/feeding and hormone secretion are responsible for the circadian modulation of the body [3]. This regulation may suffer misalignment when, for example, the sleep/wake cycle, food intake and/or physical exertion occur at times drastically different from usual [1]. Thus, one real-world condition in which circadian misalignment undoubtedly occurs is the shift work environment [4]. In addition to the adaptation of biological rhythms to inversions of periods of activity and rest, these workers are also subject to a drastic change of lifestyle, which negatively influences general health, sleep and social and family interactions [5].

Due to desynchronization of biological rhythms [6] and sleep deprivation [7], shift workers are at increased risk of developing sleep disturbances [8], and nutritional, metabolic [9], gastrointestinal and cardiac disorders [10–12], and even cancer [13], among other conditions. One way to measure circadian misalignment is by calculating the social jetlag (SJL)—a parameter that measures the discrepancy in sleep time on working days in relation to free days [14, 15]. SJL has been associated with biomarkers of inflammation and diseases such as obesity [16] and diabetes mellitus [17].

Alterations in glucose [18, 19] and lipids [20] blood levels, as well in blood pressure [21] comonly present in shift workers have been associated with erythrocyte osmotic stability, i.e., the ability of the erythrocyte to resist lysis under hyposmotic conditions. However, little is known about the influence of shift work and circadian misalignment on erythrocytes. It is known, for example, that the levels of erythropoietin (EPO), a hormone that stimulates erythrocyte production, has a well-marked circadian rhythm [22]. Moreover, it has been proposed that erythrocytes have a circadian control process that operates independently of transcriptional events, through mechanisms dependent on the oscillations of the redox system (peroxiredoxins) [23] and/or on the rhythm of potassium transport [24]. The erythrocyte is the most abundant cell in the blood, being responsible for the transport of oxygen and carbon dioxide [25]. Despite being an apparently simple and anucleate cell, the erythrocyte participates in functions that are essential to the organism, and therefore is involved in the pathophysiology of numerous diseases [20]. The osmotic fragility test is a simple way to evaluate the erythrocyte membrane behavior [26]; however, the relationship between erythrocyte membrane stability and circadian misalignment is still unclear.

Given the above understanding, the hypothesis raised in this study is that night workers have a difference in erythrocyte membrane stability in relation to day workers. Therefore, the objective of this work was to evaluate whether shift work, sleep hours and SJL are associated with biochemical, hematologic and erythrocyte membrane stability parameters.

## Material and methods

This study cross-sectional study was approved by the Ethics Committee of the Federal University of Uberlandia. Using the G*POWER application [27], the minimum sample size calculated was 78 (n), assuming a two-tailed hypothesis, a significance level (α) of 0.05, a power (1-β) of 0.85 and an effect size (Cohen's d) of 0.9. The study population comprised 79 workers men (37 night workers and 42 day workers) of two hospitals of Uberlandia, a city located in the state of Minas Gerais, Brazil. The volunteers worked in the same routine (work shift and function) for at least six months in administrative and health-related functions (nurse, physiotherapist, nursing technician, laboratory technician and stretcher bearers) and were classified according to their work shift in: 1) day workers, who worked only during the day, morning and/or afternoon, without developing any work activity at night, and with additional shifts always also daytime; and 2) night workers, who worked at least six hours after midnight, and with and without daytime additional work activities.

Individual invitations to participate in the study were made at the workplace after presentation of the study proposal to the workers. From the total of one hundred and thirty-five workers who agreed to participate in this study, a total of 79 male adult workers were selected. The exclusion criteria comprised diseases previously diagnosed and under treatment, except obesity; current or past active smoking and work schedules different from the classifications stipulated in this study. Twenty two workers refused to participate. All participants included in this study had not recently used any drug with antioxidant properties, such as vitamin C or E or acetylcysteine. Each participant signed a written informed consent.

After completing the criteria for inclusion and exclusion and selection of participants, data on socio-demographic characteristics, physical exercise and alcohol consumption were collected. All selected volunteers were subjected to anthropometric, chronobiological and sleep evaluations, and to laboratory dosages. All evaluations were performed in a single moment of the morning following a night's sleep before the work shift.

### Assessment of body composition

Anthropometric variables such as weight and height were measured, respectively, using an electronic scale (Welmy^™^) and a wall-mounted portable stadiometer e (Welmy^™^), and used to calculate the body mass index (BMI, kg/m^2^) of the volunteers [28]. The waist circumference (WC) was measured in agreement with the standard proposed by [29]. WC values of the male population studied here were considered increased when they were ≥ 94 cm [28].

### Sleep pattern, chronotype and social jetlag

These evaluations were performed by a specialized team trained in sleep studies based on the information compiled in the participants' responses to a previously reported questionnaire [30].

The bedtime on working days and rest days was obtained considering the time it took for the volunteer to fall asleep.

Sleep duration (SD), for day shift, was computed using the weighted average of self-reported sleep duration, which considers both work days and days off, using the equation:

$$SD=\frac{Self-Reported\;SD\;on\;weekdays\times 5+Self-Reported\;SD\;on\;weekends\times 2\;}{7},$$ (1)

where 5 represents the number of work days, 2 the number of days off, and 7 is the total number of days in the week.

For the night shift, SD was computed using the equation:

$$SD=\frac{Self-Reported\;SD\;on\;weekdays\times 3.5+Self-Reported\;SD\;on\;weekends\times 3.5\;}{7},$$ (2)

where 3.5 represents the number of work days and also the number of days off, and 7 is the total number of days in the week [31].

For day workers, chronotype was derived from the time of mid-sleep time on free days at the weekend (MSF) with a further correction for calculated sleep debt (MSFsc), calculated as the difference between average sleep duration at the rest days and work days (Roenneberg et al., 2007). For night workers, chronotype was calculated with the specific formula for shift workers (MSF^E^sc) proposed by Juda et al. [32]. The chronotype was classified in: early types: MSFsc ≤ 3:59 h; intermediate types: MSFsc > 4:00 and < 4:59 h; and late types: MSFsc ≥ 5:00 h [16]. Social jetlag was calculated based on the absolute difference between mid-sleep time at weekends and on weekdays [14].

### Epworth Sleepiness Scale (ESS)

Daytime sleepiness was assessed from the Epworth Sleepiness Scale (ESS)[33], previously translated into the language of the study participants [34], with a total score ≥ 8 indicating excessive drowsiness [33]. ESS is a widely used tool to characterize daytime sleepiness [35].

### Biochemical dosages

Blood collection was performed, after 8–12 h fasting in a single moment of the morning following a night's sleep before the work shift. The time of blood collection was 08:00 [08:00–08:45] a.m. for the group of day workers and 9:00 [8:00–9:45] a.m. for the group of night workers, with values expressed in median and interquartile range. This time difference was due to the fact that volunteers were instructed to maintain their normal waking hours and to sleep through all night (7–8 hours) before blood collection. Blood samples were collected only if the subjects reported that they had followed the previous guidance. The volunteers of both groups remained in the same environmental conditions, i.e., the same artificial lighting and the typical noise of the hospital environment, during this procedure. Blood samples were collected by venipuncture in tubes containing EDTA and separating gel (Vacutainer^™^, BD, Juiz de Fora, MG, Brazil) and immediately centrifuged at 1,300 x *g* for 15 min in a refrigerated centrifuge (Hitachi Koki, model CFR15XRII^™^, Hitachinaka, Japan) at 4°C. Supernatants from these centrifuges were frozen at -80°C (Panasonic^TM^, model CUK-UB2I-PW, Nijverheidsweg, Netherlands). The volunteers were instructed to fast for 12 hours and abstain from physical exercise and alcohol consumption 24 hours prior to collection. The collected blood samples were used for for determination of hematologic, biochemical and OSEM variables. The osmotic fragility test used to evaluate the OSEM variables.

### Determination of the osmotic stability of the erythrocyte membrane (OSEM)

Duplicated sets of microtubes containing 1 mL of 0.10, 0.20, 0.30, 0.40, 0.42, 0.44, 0.46, 0.48, 0.50, 0.52, 0.60, 0.70, 0.80 and 0.90 g/dL NaCl solutions were preincubated at 37 °C in a thermostated bath (Marconi^™^, Model MA 184, Piracicaba, SP, Brazil) for 10 minutes. There is no need to use a buffer because only extreme pH values, which will not be generated during lysis, can affect the process [36]. After adding 10 μL of whole blood, the tubes were carefully homogenized, reincubated at 37 °C for 30 minutes and then centrifuged at 1600 x *g* for 10 min. The amount of hemoglobin released on lysis was expressed by reading the absorbance of the supernatant at 540 nm in a UV-VIS spectrophotometer (Shimadzu^™^, UV1650TC model, Japan). Absorbance at 540 nm (A_540_) as a function of NaCl concentration was adjusted by sigmoidal regression based on the Boltzmann equation:

$$A_{540}=\frac{A_{max}\;A_{min}}{1+e^{(X-H_{50})/dX}}+A_{min},$$ (3)

where A_min_ and A_max_ are the mean absorbance values at the minimum and maximum plateaus of the simoid and represent the initial and the maximum hemolysis rate, respectively; H_50_ is the NaCl concentration capable of promoting 50% hemolysis and dX is the variation in the NaCl concentration necessary to promote 100% hemolysis [26, 37–39].

### Statistical analysis

Data analysis was performed using SPSS version 20.0 (IBM Corp., Armonk, NY). The existence of normality in the data distribution was verified using the Shapiro-Wilk test. Parametric data were presented as means and standard deviations and non-parametric data were presented as median and interquartile range. The chi-square test was used to compare variables presented in frequency. The existence of differences between the groups in relation to the studied variables (socio-demographic, anthropometric, sleep patterns, sleepiness score, chronotype, social jetlag, life habits and stress parameters) was analyzed using Student's t-test for independent samples or the Mann-Whitney test.

The Generalized Linear Model (GzLM) was used to analyze the existence of differences in biochemical parameters and membrane stability between shifts, hours of sleep, SJL and the interactions (shift x hours of sleep, shift x SJL), adjusted for age, BMI, working hours and exercise (min/wk). This statistical tool presents the data as estimated mean and Wald confidence interval (95% CI). The sequential Šidák procedure was used for the comparisons of the estimated marginal means. Statistical tests with p<0.05 were accepted as significant. The false discovery rate (FDR) through Benjamini‒Hochberg correction was used to calculate *q*-values using the *p*.*adjust* function [40].

## Results

The volunteers had aged between 21 and 65 years. Night and day workers did not differ in age (p = 0.688) and working time in the current shift (p = 0.348). The workers of the two shifts also did not present difference of weight (p = 0.427), BMI (p = 0.115) and WC (p = 334). Regarding the sleep and chronobiological variables, sleep time on days off (p = 0.170) and MSFEsc (p = 0.708) were not different between the work shifts. On the other hand, night workers presented higher workload (p<0.001), greater daytime sleepiness (p = 0.002) and shorter sleep time in workdays (p<0.001), but a higher value of social jetlag (p<0.001) in relation to the day workers (Table 1).

**Table 1:** Sociodemographic characteristics, anthropometric indices, life habits, sleep patterns, score sleepiness, chronotype and social jetlag of employees according to shift worked.

	Night (n = 37)	Day (N = 42)	p-value
**Mean Sleep Duration** (h)			
Work days	3:50 [2:22–4:27]	6:35 [5:28–7:35]	<0.001*
Rest days	7:56 ± 1:58	8:33 ± 1:52	0.170
**Chronotype (MSF**^**E**^**sc)** (h)	3:44 ± 1:00	3:38 ± 1:25	0.708
Morning	21 (56.8)	29 (69.0)	0.260
Indifferent	12 (32.4)	7 (16.7)	
Evening	4 (10.8)	6 (14.3)	
**Social Jet Lag** (h)	5:07 [2:35–7:53]	1:15 [0:45–2:02]	<0.001*
Yes	32 (86.5)	25 (59.5)	0.011*
No	5 (13.5)	17 (40.5)	
**Sleepiness Score** (Epworth)	10.76 ± 4.88	7.48 ± 4.00	0.002*
Daytime Sleepiness	16 (43.2)	9 (21.4)	0.037*
No Drowsiness	21 (56.8)	33 (78.6)	
**Age (years)**	42.43 ± 8.50	43.40 ± 12,72	0.688
**Working time** (years)	5.00 [2.00–12.5]	4.00 [2.00–10.75]	0.348
**Workload** (h/week)	57.0 [42.0–69.0]	36.0 [36.0–40.0]	<0.001*
**Anthropometry**			
Weight (kg)	83.49 ± 11.73	81.13 ± 13.97	0.427
Height (m)	1.72 ± 0.06	1.73 ± 0.07	0.585
BMI (kg/m^2^)	27.24 [26.05–29.64]	26.51 [24.00–28.49]	0.115
Waist Circumference (cm)	97.62 ± 11.04	95.17 ± 11.04	0.334
**Physical Exercise** (N, %)			
Yes	17 (45.9)	21 (50.0)	0.718
No	20 (54.1)	21 (50.0)	
Duration (min/week)	240.0 [157.50–330.00]	240.0 [135.00–435.00]	0.601
**Alcohol Consumption** (N, %)			
Yes	20 (54.1)	26 (61.9)	0.502
No	17 (45.9)	16 (38.1)	
Beer (cans/week)	4.00 [1.00–10.00]	5.0 [2.25–6.75]	0.330

Values are presented as mean ± standard deviation for normally distributed data or median (interquartile range) for non-normally distributed data, and as frequency (number, percentage) for categorical variables.

*p<0.05 indicates statistically significant difference. SJL was calculated based on the absolute difference between the average sleep time on working and rest days and was dichotomically categorized as >60 min (with SJL) or <60 min (without SJL). Comparisons between groups were done using the Student's t-test or the Mann-Whitney test, for independent samples, for data with and without normal distribution, respectively, or by the Chi-square test, for variables expressed as frequency.

There was no statistically significant difference between the work shifts in relation to the OSEM, hematologic and biochemical variables studied here (Table 2).

**Table 2:** Comparison of OSEM, hematologic and biochemical parameters between work shifts.

	Day Workers (n = 37)	Night Workers (n = 42)	p-value
A_max_ (abs)	1.24 [1.18–1.28]	1.25 [1.21–1.30]	0.207
A_min_ (abs)	0.011 ± 0,006	0.009 ± 0.007	0.400
1/H_50_ (g/dL NaCl)^-1^	2.29 [2.26–2.37]	2.31 [2.26–2.38]	0.467
dX (g/dL NaCl)	0.011 [0.009–0.013]	0.011 [0.010–0.014]	0.559
Red Blood Cells (million/mm^3^)	5.20 [4.98–5.31]	5.19 [4.85–5.41]	0.949
Hemoglobin (g/dL)	15.33 ± 1.05	15.36 ± 0.99	0.892
Hematocrit (%)	47.65 ± 3.12	47.52 ± 2.84	0.840
Mean Corpuscular Volume (fL)	91.68 ± 4.86	91.81 ± 4.70	0.911
Mean Corpuscular Hemoglobin Concentration (%)	32.2 [31.7–32.9]	32.3 [31.8–32.7]	0.836
Leukocyte Count (10^3^/mm^3^)	5.91 ± 0.15	6.05 ± 1.51	0.686
Platelet Count (10^3^/mm^3^)	213.41 ± 45.85	205.93 ± 41.70	0.450
Serum Iron (μg/dL)	114.20 [90.10–140.10]	114.5 [94.32–139.30]	0.898
Reticulocyte Count (10^3^/mm^3^)	62.37 [48.97–88.73]	56.05 [44.19–73.50]	0.205
Reticulocyte Index (%)	1.10 [1.00–1.70]	1.10 [0.90–1.50]	0.366
Total Cholesterol (mg/dL)	172.0 [155.5–198.0]	167.0 [148.25–190.25]	0.309
HDL-Cholesterol (mg/dL)	38.50 ± 6.89	39.54 ± 10.33	0.603
LDL-Cholesterol (mg/dL)	107.1 [88.75–124.80]	105.1 [87.3–125.75]	0.902
Glucose (mg/dL)	82.0 [76.0–87.5]	82.0 [76.0–87.5]	0.476
HbA1c (%)	5.3 [5.05–5.55]	5.20 [5.1–5.5]	0.437
Triglycerides (mg/dL)	127.0 [97.5–229.0]	111.0 [82.25–163.25]	0.115
Uric acid (mg/dL)	6.18 ± 1.44	6.01 ± 1.31	0.593

Abbreviations: OSEM, osmotic stability of erythrocytes membrane; A_max_, absorbance obtained by lysis of the total erythrocyte population used in the test; A_min_, absorbance obtained by hemolysis under isotonic conditions with blood; 1/H_50_, inversion of saline concentration in which there is 50% hemolysis; dX, saline concentration range involved in the lysis process of the total erythrocyte population used in the test; HbA1c, glycated hemoglobin A1c.

*p<0.05 indicates statistically significant difference. Values are presented as mean ± standard deviation for normally distributed data or median (interquartile range) for non-normally distributed data. Comparisons between groups were done using the Student's t-test or the Mann-Whitney test, for independent samples, for data with and without normal distribution, respectively.

In order to investigate the occurrence of some impropriety with the results, correlation analyzes were performed between the A_max_ variable and erythrogram variables that are conceptually associated with that OSEM variable. Strong positive associations were observed between A_max_ values and erythrocyte counts, hemoglobin levels and hematocrit values for the entire studied population (r = 0.68, p<0.001; r = 0.80, p<0.001; r = 0.71, p<0.001) and also for night (r = 0.71, p<0.001; r = 0.82, p<0.001; r = 0.67, p<0.011) and day (r = 0.64, p<0.001; r = 0.77, p<0.001; r = 0.75, p<0.001) workers, respectively. These correlations confirm the reliability of the data obtained in this study.

Table 3 presents the comparison of the studied variables in relation to the shift and average sleep time (<6 hours or ≥ 6 hours), and the interaction shift*hours of sleep, after adjustement for age, BMI, and exercise. There was an association between sleep time and variables 1/H_50_, dX, MCV, RBC count, and total and LDL-cholesterol. Considering night and day workers in a single group, and after adjustments made for age, body mass index and physical exercise, workers who slept less than 6 hours on average had higher values of 1/H50 (<6h: 2.44 [2.37–2.50]; ≥6h: 2.33 [2.28–2.38]) and dX (<6h: 0.015 [0.012–0.18]; ≥6h: 0.011 [0.009–0.013]). In addition, subjects who slept less than 6 hours had lower MCV values (<6h: 89.3 [87.1–91.5]; ≥6h: 93.6 [91.7–95.5]) and higher RBC counts (<6h: 5.33 [5.17–5.50]; ≥6h: 5.04 [4.92–5.17]) and blood levels of total (<6h: 185 [169–203]; ≥6h: 163 [151–175]) and LDL-cholesterol (<6h: 121 [107–137]; ≥6h: 98.0 [88.4–108]), in relation to those who had longer average sleep time. There was no interaction between shift and mean sleep time for any of the variables.

**Table 3:** Comparison of OSEM, hematologic and biochemical variables of shift workers in relation to the shift and average sleep time (<6 hours or ≥ 6 hours), and interaction shift*hours of sleep, after adjustement for age, body mass index, workload and physical exercise.

Parameters	Night Workers		Day Workers		Shift			Hours of sleep			Shift*Hours of sleep		
	< 6 hours of sleep (n = 23)	≥ 6 hours of sleep (n = 14)	< 6 hours of sleep (n = 9)	≥ 6 hours of sleep (n = 33)	Df	p-value	q-value	Df	p-value	q-value	Df	p-value	q-value
A_max_ (abs)	1.27 [1.22–1.31]	1.25 [1.20–1.31]	1.26 [1.21–1.32]	1.22 [1.18–1.26]	1	0.572	0.827	1	0.214	0.428	1	0.586	0.837
A_min_ (abs)	0.007 [0.004–0.013]	0.011 [0.006–0.021]	0.011 [0.005–0.022]	0.011 [0.007–0.017]	1	0.656	0.827	1	0.466	0.747	1	0.550	0.837
1/H_50_ (g/dL NaCl)^-1^	2.38 [2.28–2.47]	2.32 [2.21–2.43]	2.49 [2.37–2.62]	2.35 [2.26–2.42]	1	0.288	0.784	1	0.020*	0.1**	1	0.356	0.774
dX (g/dL NaCl)	0.014 [0.010–0.019]	0.009 [0.006–0.014]	0.016 [0.011–0.023]	0.012 [0.009–0.016]	1	0.356	0.784	1	0.031*	0.1**	1	0.769	0.849
RBC (million/mm^3^)	5.21 [5.00–5.44]	5.04 [4.78–5.30]	5.46 [5.18–5.76]	5.05 [4.86–5.24]	1	0.392	0.784	1	0.005*	0.05**	1	0.364	0.774
Hemoglobin (g/dL)	15.5 [14.8–16.2]	15.3 [14.5–16.2]	15.3 [14.4–16.2]	15.2 [14.6–15.8]	1	0.753	0.827	1	0.694	0.818	1	0.844	0.849
Hematocrit (%)	48.1 [46.2–50.1]	47.3 [45.0–49.7]	47.1 [44.7–49.6]	47.0 [45.3–48.7]	1	0.612	0.827	1	0.630	0.787	1	0.736	0.849
Mean Corpuscular Volume (fL)	92.3 [89.2–95.5]	93.9 [90.1–97.9]	86.3 [82.6–90.2]	93.2 [90.5–96.1]	1	0.114	0.784	1	0.004*	0.05**	1	0.133	0.633
Mean Corpuscular Hemoglobin Concentration (%)	32.2 [31.6–32.8]	32.3 [31.6–33.0]	32.4 [31.7–33.1]	32.4 [31.9–32.9]	1	0.786	0.827	1	0.935	0.977	1	0.830	0.849
Leukocyte Count (10^3^/mm^3^)	5.59 [4.67–6.70]	5.72 [4.54–7.22]	6.25 [4.97–7.86]	6.14 [5.24–7.21]	1	0.473	0.827	1	0.977	0.977	1	0.849	0.849
Platelet Count (10^3^/mm^3^)	223.21 [198.84–250.57]	192.94 [166.74–223.26]	212.08 [183.07–223.26]	205.69 [185.33–228.28]	1	0.938	0.938	1	0.117	0.33	1	0.387	0.774
Serum Iron (μg/dL)	139 [111–172]	124 [95.8–161]	116 [88.2–153]	112 [92.8–136]	1	0.341	0.784	1	0.486	0.747	1	0.766	0.849
Reticulocyte Index (%)	1.08 [0.86–1.34]	1.04 [0.79–1.35]	1.07 [0.81–1.40]	1.43 [1.18–1.73]	1	0.289	0.784	1	0.208	0.428	1	0.190	0.633
Total Cholesterol (mg/dL)	194 [172–220]	150 [129–176]	176 [152–206]	176 [157–196]	1	0.749	0.827	1	0.027*	0.1**	1	0.082	0.547
HDL-C (mg/dL)	38.5 [33.7–44.1]	41.8 [35.4–49.4]	38.2 [32.3–45.3]	37.8 [33.6–42.7]	1	0.565	0.827	1	0.581	0.787	1	0.557	0.837
LDL-C (mg/dL)	122 [103–144]	87.7 [70.7–109]	120 [97.7–148]	110 [94.0–127]	1	0.379	0.784	1	0.009*	0.06**	1	0.232	0.633
Glucose (mg/dL)	87.2 [81.2–93.5]	86.0 [79.0–93.6]	83.2 [76.2–90.9]	77.3 [72.7–82.2]	1	0.114	0.784	1	0.191	0.428	1	0.442	0.803
HbA1c (%)	5.35 [5.16–5.54]	5.42 [5.18–5.65]	5.32 [5.08–5.56]	5.09 [4.93–5.25]	1	0.175	0.784	1	0.372	0.676	1	0.173	0.633
Triglycerides (mg/dL)	155 [112–216]	100 [65.9–152]	96.2 [62.3–148]	137 [102–183]	1	0.708	0.827	1	0.778	0.864	1	0.050	0.547
Uric acid (mg/dL)	6.06 [5.17–7.11]	4.89 [4.03–5.95]	5.65 [4.59–6.95]	6.46 [5.60–7.44]	1	0.357	0.784	1	0.611	0.787	1	0.059	0.547

Abbreviations: OSEM, osmotic stability of erythrocytes membrane; A_max_, absorbance obtained by lysis of the total erythrocyte population used in the test; A_min_, absorbance obtained by hemolysis under isotonic conditions with blood; 1/H_50_, inversion of saline concentration in which there is 50% hemolysis; dX, saline concentration range involved in the lysis process of the total erythrocyte population used in the test; HbA1c, glycated hemoglobin A1c.

Data are presented as estimated mean and Wald confidence interval (95% CI)

*p<0.05 indicates statistically significant difference (Generalized Linear Model and sequential Šidák procedure).

q = false discovery rate of 25%.

** = significant false discovery rate.

Table 4 presents the comparison of the studied variables in relation to the shift and SJL, and the interaction shift*SJL, after adjustment for age, BMI, workload and exercise. There was a significant effect of the work shift and the SJL on the values of the stability variable A_min_, mean corpuscular hemoglobin concentration (MCHC) and the blood levels of uric acid. Considering individuals with and without SJL in a single group, and after adjustments made for age, body mass index, workload and exercise, the night workers had lower A_min_ values (0.003 [0.001–0.006]) than day workers (0.012 [0.008–0.017]); on the other hand, considering nigh and day workers in a single group, workers without SJL had lower A_min_ values (0.003 [0.001–0.006]) than workers with SJL (0.012 [0.009–0.016]). The same pattern of variation in results was also found for the parameter MCHC. Considering individuals with and without SJL in a single group, the night workers had lower MCHC values (30.8 [30.1–31.6]) than day workers (32.5 [32.1–32.8]); on the other hand, considering nigh and day workers in a single group, workers with SJL had lower MCHC values (30.9 [30.2–31.6]) than workers without SJL (32.4 [32.1–32.7]).

**Table 4:** Comparison of the studied variables in relation to the shift and social jetlag (SJL), and the interaction shift*SJL, after adjustment for age, body mass index, workload and physical exercise.

	Night Workers		Day Workers		Shift			SJL			Shift*SJL		
	With SJL (n = 33)	Without SJL (n = 4)	With SJL (n = 25)	Without SJL (n = 17)	Df	p-value	q-value	Df	p-value	q-value	Df	p-value	q-value
A_max_ (abs)	1.26 [1.22–1.30]	1.22 [1.10–1.35]	1.25 [1.20–1.30]	1.22 [1.18–1.26]	1	0.963	0.986	1	0.347	0.77	1	0.896	0.975
A_min_ (abs)	0.009 [0.006–0.013]^a^	0.001 [0.0002–0.004]^b^	0.016 [0.009–0.027]^a^	0.009 [0.006–0.014]^a^	1	0.003*	0.03**	1	<0.001*	<0.001**	1	0.034*	0.34
1/H_50_(g/dL NaCl)^-1^	2.36 [2.29–2.44]	2.38 [2.13–2.65]	2.28 [2.18–2.38]	2.45 [2.37–2.53]	1	0.926	0.986	1	0.201	0.67	1	0.279	0.507
dX (g/dL NaCl)	0.013 [0.009–0.017]	0.012 [0.005–0.028]	0.009 [0.007–0.013]	0.015 [0.012–0.020]	1	0.972	0.986	1	0.438	0.79	1	0.264	0.507
Red Blood Cells (million/mm^3^)	5.14 [4.94–5.35]	5.14 [4.50–5.87]	5.08 [4.82–5.35]	5.21 [5.00–5.43]	1	0.986	0.986	1	0.733	0.91	1	0.712	0.89
Hemoglobin (g/dL)	15.4 [14.8–16.0]	14.5 [12.7–16.4]	15.6 [14.8–16.4]	15.0 [14.5–15.6]	1	0.561	0.986	1	0.160	0.67	1	0.671	0.89
Hematocrit (%)	47.6 [46.0–49.2]	49.3 [44.0–55.2]	48.0 [45.9–50.2]	46.3 [44.7–47.9]	1	0.482	0.986	1	0.980	0.91	1	0.267	0.507
Mean Corpuscular Volume (fL)	92.6 [89.8–95.4]	96.1 [86.8–106]	94.7 [91.1–98.5]	89.0 [86.3–91.8]	1	0.435	0.986	1	0.651	0.91	1	0.080	0.507
Mean Corpuscular Hemoglobin Concentration (%)	32.4 [31.9–32.8]^a^	29.3 [28.1–30.6]^b^	32.4 [31.9–33.0]^a^	32.5 [32.0–32.9]^a^	1	<0.001*	<0.001**	1	<0.001*	<0.001**	1	<0.001*	<0.001**
Leukocyte Count (10^3^/mm^3^)	5.60 [4.82–6.50]	4.48 [2.70–7.42]	7.01 [5.76–8.53]	5.75 [4.92–6.72]	1	0.166	0.664	1	1.36	1.36	1	0.927	0.976
Platelet Count (10^3^/mm^3^)	214.60 [193.50–238.01]	184.23 [130.89–259.29]	195.11 [170.44–223.37]	211.49 [190.35–234.98]	1	0.856	0.986	1	0.705	0.91	1	0.223	0.507
Serum iron(μg/dL)	133 [110–161]	128 [68.5–239]	111 [86.8–143]	113 [93.9–137]	1	0.486	0.986	1	0.947	0.91	1	0.862	0.976
Reticulocyte Index (%)	1.07 [0.88–1.30]	0.88 [0.46–1.67]	1.35 [1.05–1.70]	1.30 [1.06–1.58]	1	0.160	0.664	1	0.506	0.79	1	0.663	0.89
Total Cholesterol (mg/dL)	179 [160–202]	142 [96.8–209]	172 [148–200]	173 [154–195]	1	0.553	0.986	1	0.279	0.705	1	0.274	0.507
HDL-C (mg/dL)	40.4 [36.1–45.1]	33.3 [23.0–48.1]	35.2 [30.5–40.6]	40.2 [35.9–45.0]	1	0.840	0.986	1	0.774	0.91	1	0.112	0.507
LDL-C (mg/dL)	109 [93.2–129]	87.4 [51.0–149]	108 [87.7–133]	111 [94.9–132]	1	0.532	0.986	1	0.514	0.79	1	0.384	0.64
Glucose (mg/dL)	86.9 [81.8–92.4]	74.7 [61.2–91.3]	78.9 [72.9–85.4]	79.5 [74.7–84.5]	1	0.792	0.986	1	0.195	0.67	1	0.157	0.507
HbA1c (%)	5.40 [5.20–5.54]	5.16 [4.65–5.73]	5.19 [4.99–5.41]	5.15 [4.99–5.32]	1	0.633	0.986	1	0.419	0.79	1	0.602	0.976
Triglycerides (mg/dL)	134 [100–179]	100 [38.3–265]	143 [98.1–208]	106 [78.5–142]	1	0.864	0.986	1	0.282	0.705	1	0.983	0.983
Uric Acid (mg/dL)	5.68 [4.94–6.53]	3.62 [2.29–5.74]	6.45 [5.39–7.73]	5.96 [5.17–6.87]	1	0.048*	0.32	1	0.039*	0.26	1	0.149	0.507

Abbreviations: A_max_, absorbance obtained by lysis of the total erythrocyte population used in the test; A_min_, absorbance obtained by hemolysis under isotonic conditions with blood; 1/H_50_, inversion of saline concentration in which there is 50% hemolysis; dX, saline concentration range involved in the lysis process of the total erythrocyte population used in the test; HbA1c, glycated hemoglobin A1c.

Data are presented as estimated mean and Wald confidence interval (95% CI).

^a,b^ Different letter pairs indicate statistically significant differences (p<0.05) between pairs of subgroups (Generalized Linear Model).

*p<0.05 indicates statistically significant difference. SJL was calculated based on the absolute difference between the average sleep time on working and rest days and was dichotomically categorized as > 60 min (with SJL) or < 60 min (without SJL) (Generalized Linear Model and sequential Šidák procedure).

q = false discovery rate of 25%.

** = significant false discovery rate.

## Discussion

The present study evaluated whether sleep time, SJL and shift work are associated with parameters of erythrocyte membrane stability. The use of the Generalized Linear Model and sequential Šidák procedure showed that workers without SJL presented higher membrane stability, since they presented lower A_min_ values, in relation to workers with SJL. On the other hand, the use of this same statistical formalism showed that workers who have less than 6 hours of sleep have erythrocytes with greater osmotic stability, since they presented higher values of 1/H_50_ and dX. It is important to note that this behavior, although evaluated *in vitro*, reflects changes present *in vivo* conditions of the workers.

**Fig 1:**
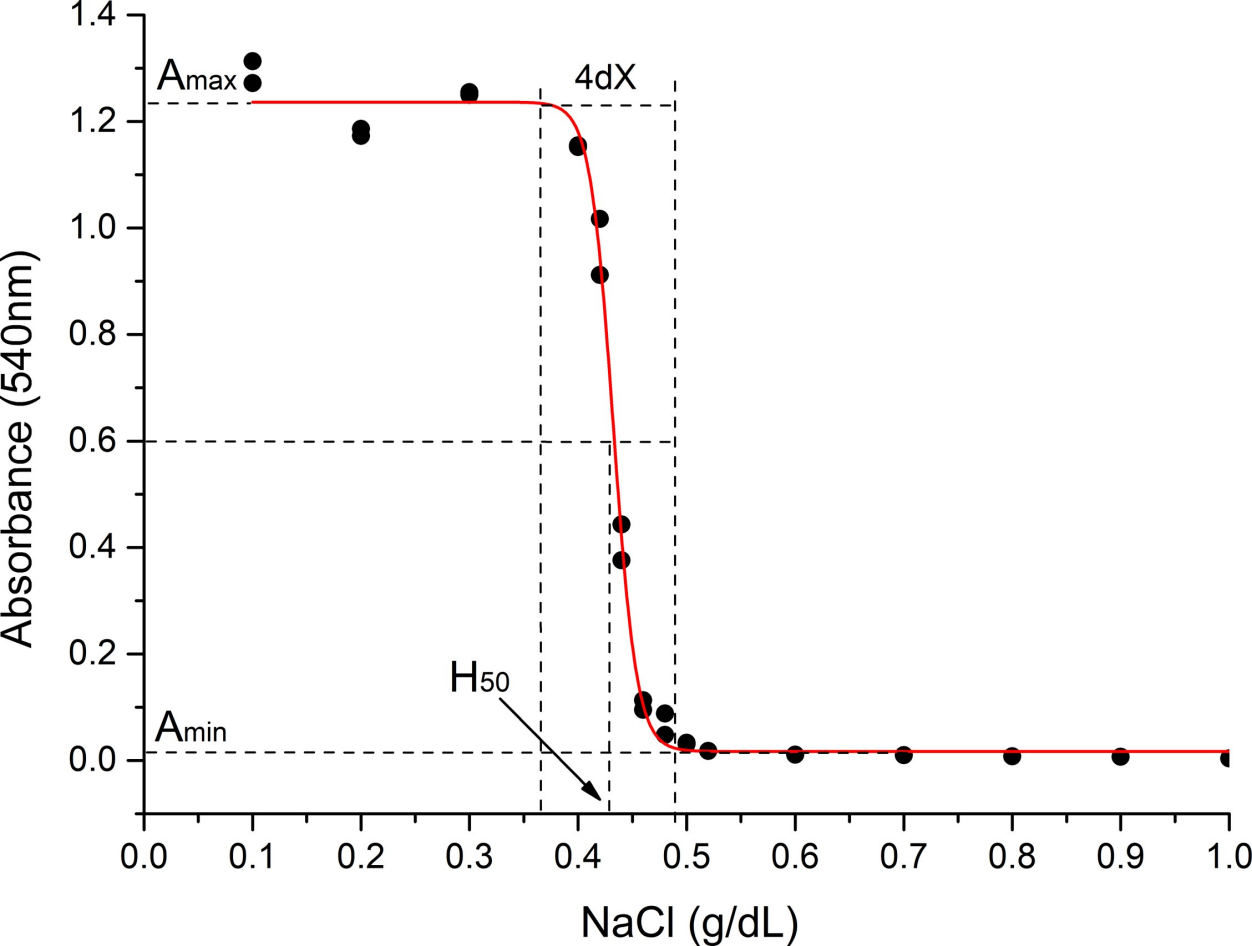
Boltzmann's sigmoidal fitting of a typical curve erythrocyte lysis in relation to NaCl concentration in a shift worker volunteer. H_50_ is the NaCl concentration that promotes 50% hemolysis; dX is the salt concentration variation necessary to promote ¼ of the total hemolysis; A_min_ is the minimum value of absorbance presented under conditions of blood-like osmolarity; and A_max_ is the maximum value of absorbance associated with 100% hemolysis.

It is also important to note that A_min_, 1/H_50_ and dX, which are all erythrocyte membrane stability variables, have different meanings. The understanding of the meanings of those parameters depends on a more detailed analysis of Fig 1. In this figure, absorbance values measured at 540 nm in the osmotic stability test represent the free hemoglobin content in the medium. The basic task of the erythrocyte osmotic stability assay is to submit a given population of erythrocytes present in a blood sample to environments of decreasing osmolarity determined by the decrease in NaCl (X) concentration. As can be seen, A_min_ is a variable that is directly proportional to the initial lysis rate, even under conditions of blood-like osmolarity. Therefore, this variable is the one that most represents the membrane stability of erythrocytes *in vivo*. Lower A_min_ values mean that erythrocytes are more stable under blood-like osmolarity conditions. On the other hand, as can still be seen in Fig 1, H_50_ and dX are variables that in fact will represent the osmotic behaviour of RBC. Indeed, it is easy to infer from that figure that H_50_ is a variable of osmotic fragility, since higher values of H_50_ mean less osmotic stability of erythrocytes, while dX is effectively a variable of osmotic stability, since higher values of dX mean the need for a greater decrease in the saline concentration of the medium to promote lysis of the whole erythrocytes population used in the assay. That’s why the H_50_ results are often represented as 1/H_50_, so that both 1/H_50_ and dX can represent the osmotic stability of those cells [41].

The erythrocyte membrane stability may be influenced by several factors, such as the LDL-C levels [26], which were also investigated in this study. Indeed, the cholesterol content of the erythrocyte membrane is directly associated with LDL-C levels, as a function of cholesterol transfer from this plasma lipoprotein to the red blood cell membrane [42, 43]. It is important to note that this increase in membrane cholesterol content to a certain extent allows the membrane to achieve the so-called critical fluidity and the stability required to perform its functions. Thus, the cholesterol content in the membrane is important to maintain erythrocyte stability. That is why non-excessive increases in membrane cholesterol content raise the osmotic stability of the cell [44]. However, beyond this critical level, the increase in cholesterol content may decrease membrane stability, due to a decrease in the deformability, essential property to allow the passage of the erythrocyte through capillaries of narrower caliber by the organism [20, 45, 46], leading to impairment of its functions. In the present study, since erythrocytes of workers who had less than six hours of sleep per day were osmotically more stable, it is possible that this increased erythrocyte stability was due to higher levels of LDL-C, since those workers also had higher levels of LDL-C (Table 3). This makes a lot of sense with the recognized influence that plasma lipid concentrations suffer from circadian misalignment and sleep deprivation [47], especially with the inverse association reported between blood levels of non-HDL-C and sleep duration [48].

The occurrence of association of sleep deprivation and SJL with erythrocyte membrane stability mediated by the lipid profile is a very relevant issue, since erythrocyte dysfunction may represent one of the mechanisms by which the existence of dyslipidemia can influence cerebrovascular-vascular health. This makes a lot of sense, not only because the red blood cells are agents of oxygen transport in the circulation, but also because erythrocytes with excess membrane cholesterol appear to play a deleterious role in the progression of atherosclerosis [20].

Regarding the two stability variables that had a relation with sleep time, it is important to highlight dX. An extremely small value of dX indicates a transition between two states, one intact and another lysate, ie, an all-or-nothing transition, while a higher value of dX indicates existence of intermediate states, which suggests existence of cellular heterogeneity in the lysis transition, with subpopulations of erythrocytes with different vulnerabilities to osmotic lysis.

Among the erythrogram parameters, the red cell distribution width (RDW) has a meaning similar to dX, since RDW, which is is obtained from the standard deviation of the mean corpuscular volume (MCV), represents the volume variability of the erythrocyte. This is why RDW is widely used to indicate heterogeneity in the erythrocyte population. Certainly it is because of the common meaning between dX and RDW that these two variables have been directly associated [26]. Unfortunately, in the present study there was no evaluation of RDW, but the association of higher dX values with an average sleep time < 6 h suggests the existence of greater variability in the erythrocyte population in this group. This question is very important because RDW was associated with sleep time according to a U-shaped curve, with lower RDW values associated with mean sleep duration of 7–8 h and higher values associated with sleep duration time ≤ 5 and ≥10 hours per night. This is very worrying, since the elevation of RDW has the potential to predict premature mortality [49] due to many diseases [50–52], among which the cardiovascular disease of atherosclerotic origin stands out [53–59], particularly by the interposition of the erythrocyte in the relationship between hypercholesterolemia and progression of the atherosclerotic lesion [20]. Surely this is an important reason why the shift work has been considered as a risk factor for hypertension, inflammation and cardiovascular disease [60, 61].

The association between A_min_ and SJL also deserves attention, bearing in mind that A_min_ has a different meaning in relation to the other stability variables, as previously mentioned. In this case, the fact that workers without SJL have presented higher membrane stability, since they have lower A_min_ values in relation to workers with SJL, seems to be a good association for the shift workers without SJL. The meaning of this decrease in A_min_ may be associated with decreased MCHC, since the individuals without SJL had lower values of A_min_ and MCHC in relation to the others. Indeed, erythrocytes with lower hemoglobin concentrations should be under the influence of lower osmotic pressure and, when lysed, will release less hemoglobin. SJL has been associated with inflammatory biomarkers [17] and in this study was positively associated with the amount of lymphocytes (r = 0.23, p <0.05). Actually, in addition to SJL, sleep restriction has also been related to changes in immune function, with a gradual increase in subpopulations of white blood cells and changes in their rhythymicity [62].

In addition to sleep deprivation [7], shift work causes misalignment of the biological rhythms, which can negatively affect the regulation of the hematologic system [63]. The erythrocyte population in the blood is under constant change due to the removal of degenerate and/or old erythrocytes and the production of new erythrocytes [64]. Circulating erythrocytes exhibit circadian fluctuation that is regulated not only by the endogenous biological clock, but also by the light/dark and sleep/wake cycles [65, 66]. The subcellular processes within the erythrocytes are not the only ones that undergo circadian oscillations; the number of red blood cells also varies rhythmically [65]. The higher erythrocyte counts and lower MCV values observed in the group of workers who slept on average less than 6 hours per day may involve some regulatory mechanism, certainly associated with circadian changes in erythropoietin levels [22], so that without affecting hemoglobin levels the production of a greater quantity of erythrocytes would mean the generation of erythrocytes with lower hemoglobin concentrations and lower MCV values. However, the mechanism by which this influence would have occurred can not be inferred from the results of this study. On the other hand, the lower MCV values and the higher RBC counts in the group of workers who slept on average less than 6 hours per day may also be due to the stochastic influence of the lower average sleep time, caused by shift work, and involve aspects of selective nutritional nature, since erythropoiesis and erythrogram are influenced by several nutritional factors. But the present study did not evaluate the blood levels of nutritional factors whose alterations could support this hypothesis.

It is important that other limitations of the present study are also considered. First, the cross-sectional nature of the study does not allow the establishment of causal relationships. Second, the relatively small population size impairs its characterization and analysis, although significant results have been found in this study. Another difficulty found in the study was the variability of the workload between the two work shifts, which required statistical adjustments to remove the effect of this and other confounding factors. Therefore, it is important to highlight the need to carry out new studies using new tools in order to clarify how the circadian rhythm can influence the erythrocyte membrane stability, leading to a new understanding of its outcomes in the health of these workers. There is also a need to study further the relation between shift work and chronotype, with the aim to reduce the consequences of circadian rhythm misalignment and sleep deprivation [67, 68]. It is also necessary to emphasize that the use of subjective methods of analysis, such as the questionnaire used for sleep evaluation, a method that depends on memory and motivation of the participants, rather than the use of objective methods such as actigraphy, constitutes a limitation of this study. In addition, the exclusion of female workers also contributes to limit the impact of the results of the present study. It is also essential to emphasize that the small size of the subgroups after stratification of the groups of workers by hours of sleep and the presence of social jetlag also represents a fragility of this study.

In summary, the results of this study confirm the initial hypothesis raised, since workers with SJL have erythrocytes with lower stability compared to workers without SJL, under conditions of blood-like osmolarity. On the other hand, regardless of the work shift, sleeping on average less than 6 hours per day is associated with *in vitro* increase in erythrocyte membrane stability and cellular heterogeneity. To the best of our knowledge, this is the first study that has identified that a sleep time < 6 hours per day is able to interfere with the erythrocyte membrane stability. It is possible that this behavior is associated with changes in the lipid profile. Due to the high risk of diseases in this group, the execution of new studies focusing on hematologic parameters in shift workers becomes a task of great importance.

## Supporting information

S1 File The database file for this manuscript. (XLSX)
